# A Functional Relationship Between UNC45A and MYO5B Connects Two Rare Diseases With Shared Enteropathy

**DOI:** 10.1016/j.jcmgh.2022.04.006

**Published:** 2022-04-11

**Authors:** Qinghong Li, Zhe Zhou, Yue Sun, Chang Sun, Karin Klappe, Sven C.D. van IJzendoorn

**Affiliations:** Department of Biomedical Sciences of Cells and Systems, Section Molecular Cell Biology, University of Groningen, University Medical Center Groningen, Groningen, the Netherlands

**Keywords:** UNC45A, MYO5B, myosin Vb, microvillus inclusion disease, chaperone, osteo-oto-hepato-enteric syndrome, CAS, CRISPR-associated protein, CRISPR, clustered regularly interspaced short palindromic repeats, KO, knockout, MVID, microvillus inclusion disease, O2HE, osteo-oto-hepato-enteric, RAB, rat sarcoma-associated binding protein, RE, recycling endosomes, SD, standard deviation, UNC45, uncoordinated 45, WT, wild-type

## Abstract

**Background & Aims:**

UNC45A is a myosin (co-)chaperone, and mutations in the *UNC45A* gene were recently identified in osteo-oto-hepato-enteric (O2HE) syndrome patients presenting with congenital diarrhea and intrahepatic cholestasis. Congenital diarrhea and intrahepatic cholestasis are also the prime symptoms in patients with microvillus inclusion disease (MVID) and mutations in *MYO5B*, encoding the recycling endosome–associated myosin Vb. The aim of this study was to determine whether UNC45A and myosin Vb are functionally linked.

**Methods:**

CRISPR-Cas9 gene editing and site-directed mutagenesis were performed with intestinal epithelial and hepatocellular cell lines, followed by Western blotting, quantitative polymerase chain reaction, and scanning electron and/or confocal fluorescence microscopy to determine the relationship between (mutants of) UNC45A and myosin Vb.

**Results:**

UNC45A depletion in intestinal and hepatic cells reduced myosin Vb protein expression, and in intestinal epithelial cells, it affected 2 myosin Vb-dependent processes that underlie MVID pathogenesis: rat sarcoma-associated binding protein (RAB)11A-positve recycling endosome positioning and microvilli development. Reintroduction of UNC45A in UNC45A-depleted cells restored myosin Vb expression, and reintroduction of UNC45A or myosin Vb, but not the O2HE patient *UNC45A*-c.1268T>A variant, restored recycling endosome positioning and microvilli development. The O2HE patient-associated p.V423D substitution, encoded by the *UNC45A*-c.1268T>A variant, impaired UNC45A protein stability but as such not the ability of UNC45A to promote myosin Vb expression and microvilli development.

**Conclusions:**

A functional relationship exists between UNC45A and myosin Vb, thereby connecting 2 rare congenital diseases with overlapping enteropathy at the molecular level. Protein instability rather than functional impairment underlies the pathogenicity of the O2HE syndrome–associated UNC45A-p.V423D mutation.

## Graphical abstract

**Figure:**
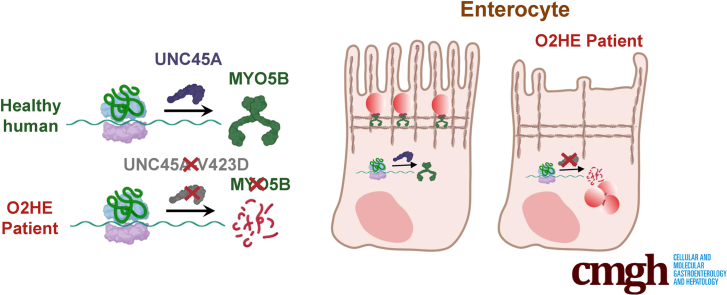

**Summary**

Two genes in which mutations are associated with different human diseases with shared intestinal symptoms are shown to be functionally linked, thereby connecting these diseases at the molecular level. The pathogenic mechanism of one of the patient’s mutations was demonstrated.

Uncoordinated (UNC)-45 belongs to the conserved UNC-45/Cro1/She4p (UCS) family of myosin (co-)chaperones.^1^, ^2^, ^3^, ^4^ Studies in nematodes, clawed frogs, zebrafish, and fruit flies demonstrated that UNC45 acts as a myosin-specific chaperone, promoting the folding of the myosin adenosine triphosphatase domain. Whereas invertebrates have one *UNC45* gene, vertebrates have two *UNC45* gene homologues, *UNC45A* and *UNC45B*. The human *UNC45B* gene (located on chromosome 17) is predominantly expressed in muscle cells, whereas the human *UNC45A* gene (located on chromosome 15) is ubiquitously expressed (https://www.proteinatlas.org/ENSG00000140553-UNC45A/tissue).

Bi-allelic compound heterozygous mutations in the *UNC45A* gene were reported in 4 patients of 3 families who clinically presented with congenital (secretory) diarrhea, cholestasis, sensorineural hearing loss, and/ or bone fragility, referred to as osteo-oto-hepato-enteric (O2HE) syndrome (Online Mendelian Inheritance in Men #619377).^5^ Muscle weakness, which is the primary symptom of congenital myopathy caused by *UNC45B* mutations,^6^ was not reported in O2HE patients. Seven unique disease-associated *UNC45A* variants were identified that affected amino acid residues across the UNC45A protein.^5^ Protein analyses in O2HE patient cells showed that these mutations resulted in 70%–90% reduction in the expression of the UNC45A protein.^5^ Liver biopsies of 2 of 3 patients who presented with cholestasis showed mislocalization of a cholestasis-associated bile acid transporter in hepatocytes, which resolved at later age.^5^ Duodenal biopsies of 3 of the 4 patients who presented with diarrhea were reported to show partial, mild villus atrophy and/or brush border abnormalities.^5^ The cellular mechanisms that underlie the congenital diarrhea and cholestasis in O2HE patients are not understood.

Notably, symptoms displayed by O2HE patients, except for bone fragility, have also been reported as direct consequences of mutations in myosin-encoding genes. Indeed, inherited non-syndromic sensorineural hearing loss is a prime symptom of patients with *MYO1A*, *MYO6*, *MYO7A, MYO15,* or *MYH9* mutations.^7^ Diarrhea and/or cholestasis are the prime symptoms of patients diagnosed with microvillus inclusion disease (MVID) (Online Mendelian Inheritance in Men #251850) and/or familial intrahepatic cholestasis and carrying bi-allelic *MYO5B* mutations.^8^, ^9^, ^10^ Moreover, *MYO5B* mutations have been shown to cause the mislocalization of bile acid transporters in the hepatocytes of some patients^9^, ^10^, ^11^ and villus atrophy and brush border abnormalities in the small intestine.^8^^,^^12^^,^^13^ Conceivably, loss of UNC45A expression may give rise to symptoms through disabling the function of these myosin proteins.

In this study we focused on the relationship between UNC45A and the intestinal symptoms. Of the unconventional human myosins, thus far only the myosin IIa protein has been demonstrated to be a client for UNC45A.^14^ However, patients with mutations in the myosin IIa-encoding *MYH9* gene do not develop diarrhea, and reciprocally, O2HE patients do not develop macrothrombocytopenia (a clinical hallmark of *MYH9*-related disease). Also, patients with inherited sensorineural deafness due to mutations in the various other myosin-encoding genes do not typically develop diarrhea. Defects in these deafness-related myosins are therefore not likely to underlie the intestinal symptoms in patients with O2HE. Bone fragility and diarrhea also do not appear to be strongly correlated in O2HE because one O2HE patient with diarrhea did not present with bone fragility, and one O2HE patient with bone fragility did not present with diarrhea.^5^

Yeast-two-hybrid experiments indicated that UNC45 may interact with the motor domain of Hum2 (the *Caenorhabditis elegans* orthologue of myosin V).^15^ She4p, which is the budding yeast orthologue of UNC45, was shown to interact with type V myosins, and loss of She4p caused the mislocalization of the type V myosin Myo4p in *Saccharomyces cerevisiae*.^16^ Furthermore, in the vertebrate zebrafish (*Danio rerio*), depletion of either intestinal *unc45a*^5^ or the *MYO5B* orthologue *goosepimples*^17^ resulted in a defective development of intestinal folds. The overlap in intestine-related symptoms and reported overlap in intestinal tissue abnormalities between O2HE patients and MVID patients may suggest a functional relationship between UNC45A expression and myosin Vb, which thus far has not been studied. Therefore, the aim of this study was to investigate whether a functional link exists between UNC45A and myosin Vb.

## Results

### Loss of UNC45A Caused a Reduction in Myosin Vb Protein Expression

**Figure 1:**
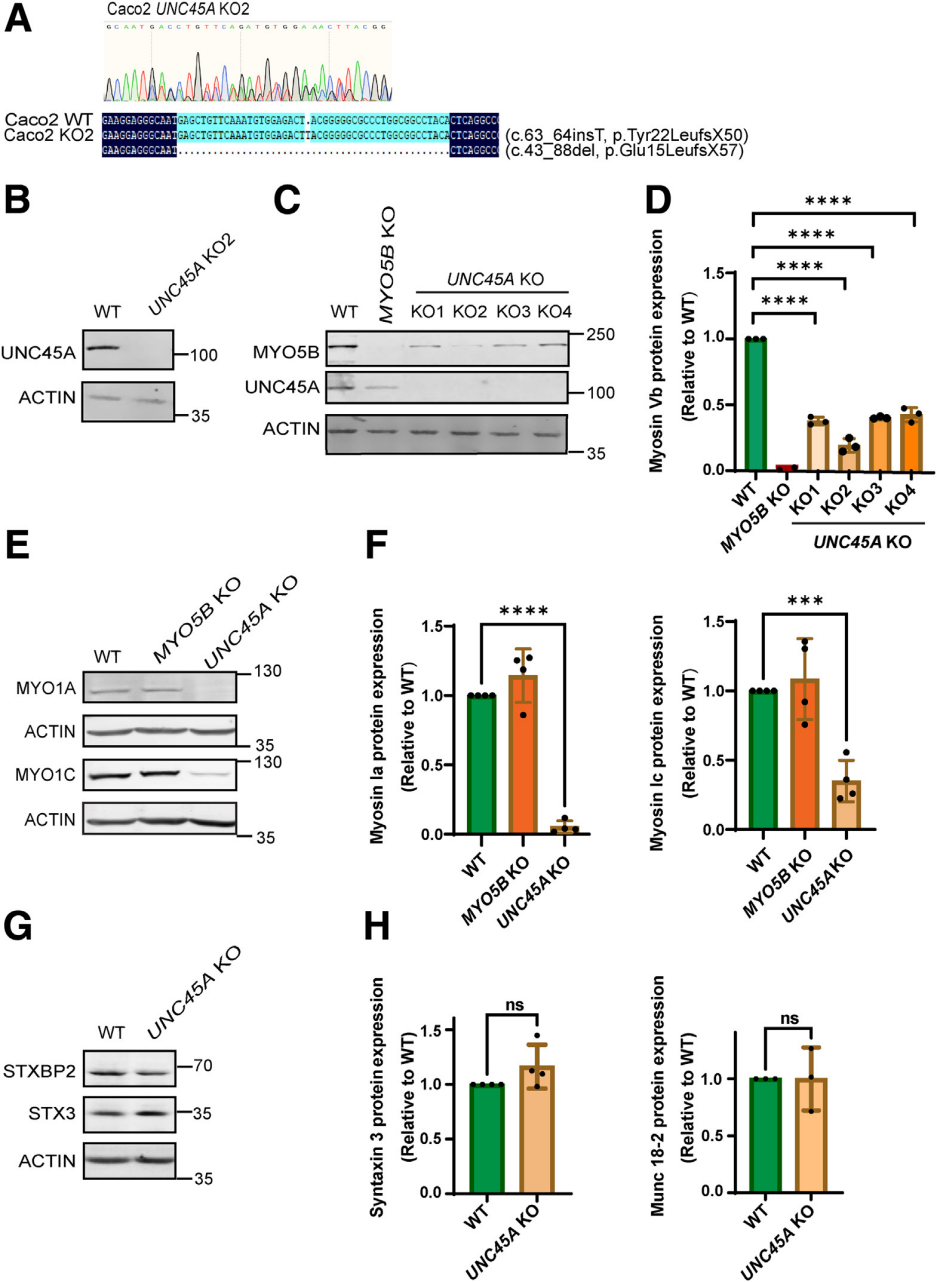
**Effect of loss of UNC45A expression on expression of myosin Vb, myosin 1A, myosin 1C, syntaxin-3, and munc18-2 in Caco-2 cells.** (*A*) Sequencing chromatogram of DNA fragment targeted by CRISPR-Cas9 in Caco-2 *UNC45A* KO2 cells. Double peaks indicate heterozygous compound mutations, and their sequences aligned to WT sequence. Software Chromas and DNAMAN are used to analyze sequencing results. (*B*) Western blot for UNC45a in Caco-2 WT and Caco-2 *UNC45A* KO2 cells. (*C*) Representative Western blot for myosin Vb and UNC45A in Caco-2 WT, Caco-2 *MYO5B* KO, and 4 different Caco-2 *UNC45A* KO cell lines. (*D*) Quantification of relative myosin Vb expression by Western blot in Caco-2 WT, Caco-2 *MYO5B* KO, and 4 different Caco-2 *UNC45A* KO cell lines. (*E*) Representative Western blot for MVID-associated syntaxin-3 and munc18-2 in Caco-2 WT and Caco-2 *UNC45A* KO cell lines. (*F*) Quantification of relative syntaxin-3 and munc18-2 expression levels by Western blot in Caco-2 WT and Caco-2 *UNC45A* KO cell lines. (*G*) Representative Western blot showing the fefcts of UNC45A depletion on the experession levels of STXBP2 (munc18-2) and STX (syntaxin-3) in Caco-2 cells. Actin was used as loading control. (*H*) Quantification of the Western blots as depicted in panel G. N ≥ 3 independent experiments. Error bars indicate mean ± standard deviation (SD). *Black dots* indicate the individual data points. *t* test: ∗∗∗∗*P* < .0001.

To investigate a functional relationship between UNC45A and myosin Vb, we used the clustered regularly interspaced short palindromic repeats (CRISPR) and CRISPR-associated protein (Cas)9–based technology to knockout the *UNC45A* gene in human colon adenocarcinoma Caco-2 cells. Caco-2 cells are widely used model cell lines for the study of intestinal epithelial cell biology and as cell models for inherited congenital diarrheal disorders including MVID.^8^^,^^13^^,^^18^^,^^19^ *UNC45A* sequence analyses in the treated cell lines revealed compound heterozygous frameshift mutations (Tyr22LeufsX50 and Glu15LeufsX57) in the *UNC45A* genes in Caco-2 cells (Figure 1*A*). These frameshift mutations were predicted to result in premature termination codons and subsequent loss of expression of the UNC45A protein. Western blot analyses confirmed the absence of the UNC45A protein when compared with control cells (Figure 1*B*).

*UNC45A* knockout (KO) Caco-2 cells (4 independent clones) showed a reduced expression of the myosin Vb protein when compared with control cells, as evidenced by Western blot (Figure 1*C* and *D*). The specificity of the myosin Vb antibody was validated in *MYO5B* KO cells (Figure 1*C*). Quantification of Western blots of 3 independent experiments revealed 60%–80% reduction in full-length myosin Vb protein expression in Caco-2 *UNC45A* KO cells (Figure 1*D*). Occasionally a reduction in UNC45A expression in *MYO5B* KO cells was observed (Figure 1*C*), but this was not statistically significant over multiple independent experiments, suggesting that the relationship between UNC45A and myosin Vb expression is unidirectional. The effects of UNC45A depletion were not limited to myosin Vb protein expression because also a reduced expression of myosin 1A and 1C was observed (Figure 1*E* and *F*). The expression levels of 2 other gene products that have been associated with MVID, ie, the *STX3*- and *STXBP2*-encoded syntaxin-3^20^, ^21^, ^22^ and munc18-2^22^, ^23^, ^24^ proteins, respectively, were unaffected by the loss of UNC45A expression (Figure 1*G* and *H*).

**Figure 2:**
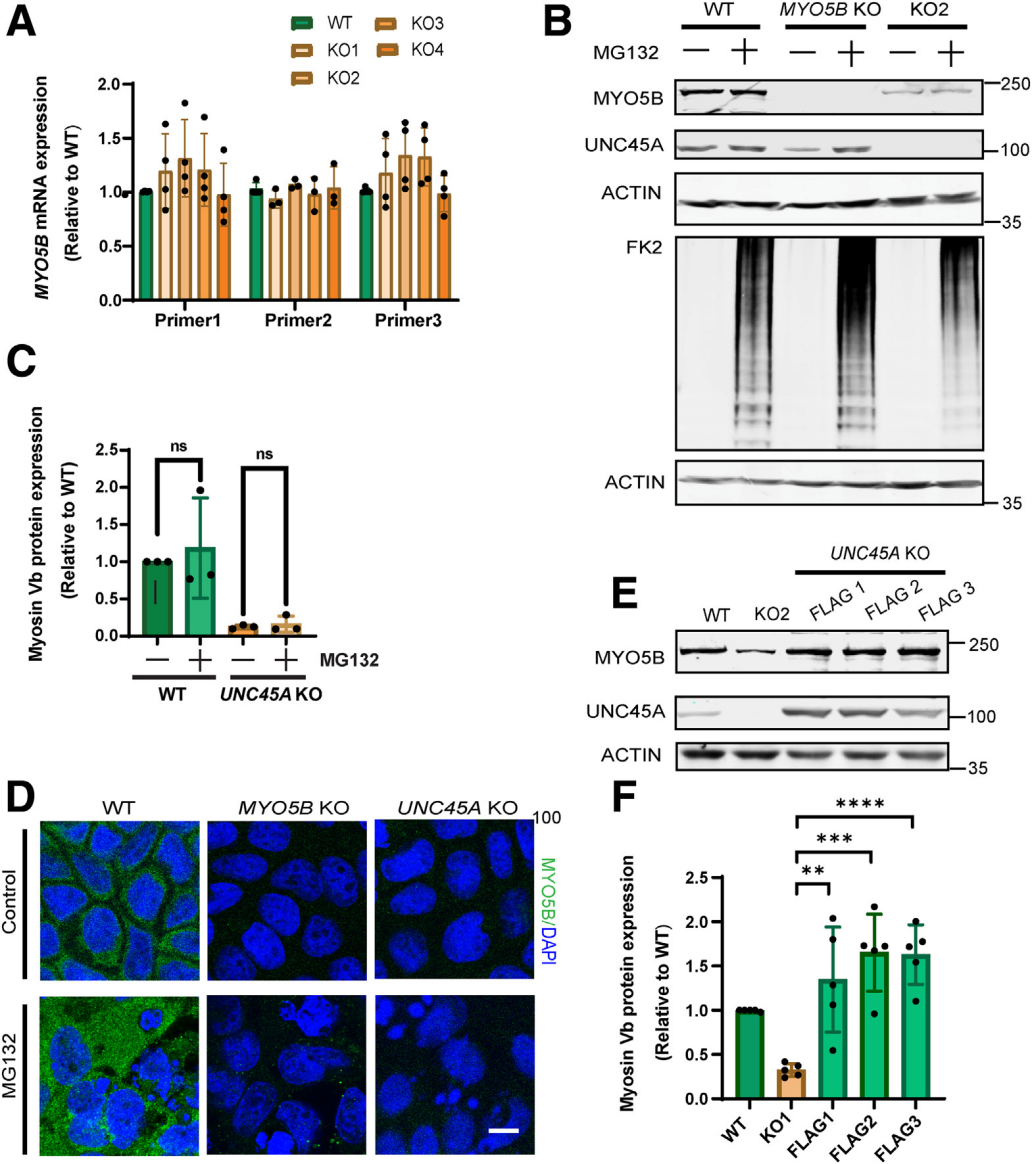
**Loss of UNC45A caused a reduction in myosin Vb protein expression.** (*A*) Quantification of relative mRNA expression of *MYO5B* gene in Caco-2 WT and 4 different Caco-2 *UNC45A* KO cell lines. (*B*) Representative Western blot showing expression of myosin Vb, unc45a, FK2 (UBCJ2) in Caco-2 WT, Caco-2 *MYO5B* KO, and Caco-2 *UNC45A* KO cells and treated with MG132 (5 μmol/L, 16 hours) respectively. (*C*) Quantification of Western blots of which a representative blot is shown in (*B*). (*D*) Immunofluorescence microscopy image of endogenous myosin Vb in WT, *UNC45A* KO2 cells, and treated with MG132 (5 μmol/L, 16 hours), respectively. Scale bar, 10 μm. (*E*) Western blot showing the expression level of myosin Vb in Caco-2 WT and 3 UNC45A-FLAG re-expression *UNC45A* KO2 cell lines. Two clones are constructed by primers Myc Deletion forward1 and reverse1; another clone was constructed by Myc Deletion forward 2 and reverse2. (*F*) Quantification of myosin Vb expression by Western blot in Caco-2 WT, Caco-2 *UNC45A* KO, and 3 different UNC45A-FLAG re-expression cell lines. (*C* and *F*) N ≥ 3 independent experiments. Error bars indicate mean ± SD. *Black dots* indicate individual data points. *t* test, ∗∗∗∗*P* < .0001.

Quantitative polymerase chain reaction (using 3 different primer sets) revealed no reduction in myosin Vb mRNA in *UNC45A* KO cells when compared with control cells (Figure 2*A*). Treatment of *UNC45A* KO cells with the peptide aldehyde Cbz-leu-leu-leucinal (MG132), which is a potent proteasome inhibitor, caused an increase in the amount of ubiquitinylated proteins as evidenced by the increased reactivity toward the anti-ubiquitinylated proteins antibody FK2 (demonstrating its activity) but did not restore expression of full-length myosin Vb protein (Figure 2*B* and *C*), suggesting that myosin Vb was not synthesized in UNC45A depleted cells. This was confirmed by immunofluorescence microscopy with antibodies against myosin Vb (Figure 2*D*).

The reintroduction of human UNC45A carrying a FLAG (DYKDDDDK)-tag at its carboxyl-terminus (UNC45A-FLAG) in *UNC45A* KO Caco-2 cells (3 independent clones) restored myosin Vb expression when compared with untreated *UNC45A* KO cells and wild-type (WT) cells (Figure 2*E* and *F*). Together, the data unequivocally demonstrate that loss of UNC45A caused a reduction in myosin Vb protein expression.

### Loss of UNC45A Caused a Microtubule-Dependent Redistribution of RAB11A-Positive Recycling Endosomes in Caco-2 Cells

**Figure 3:**
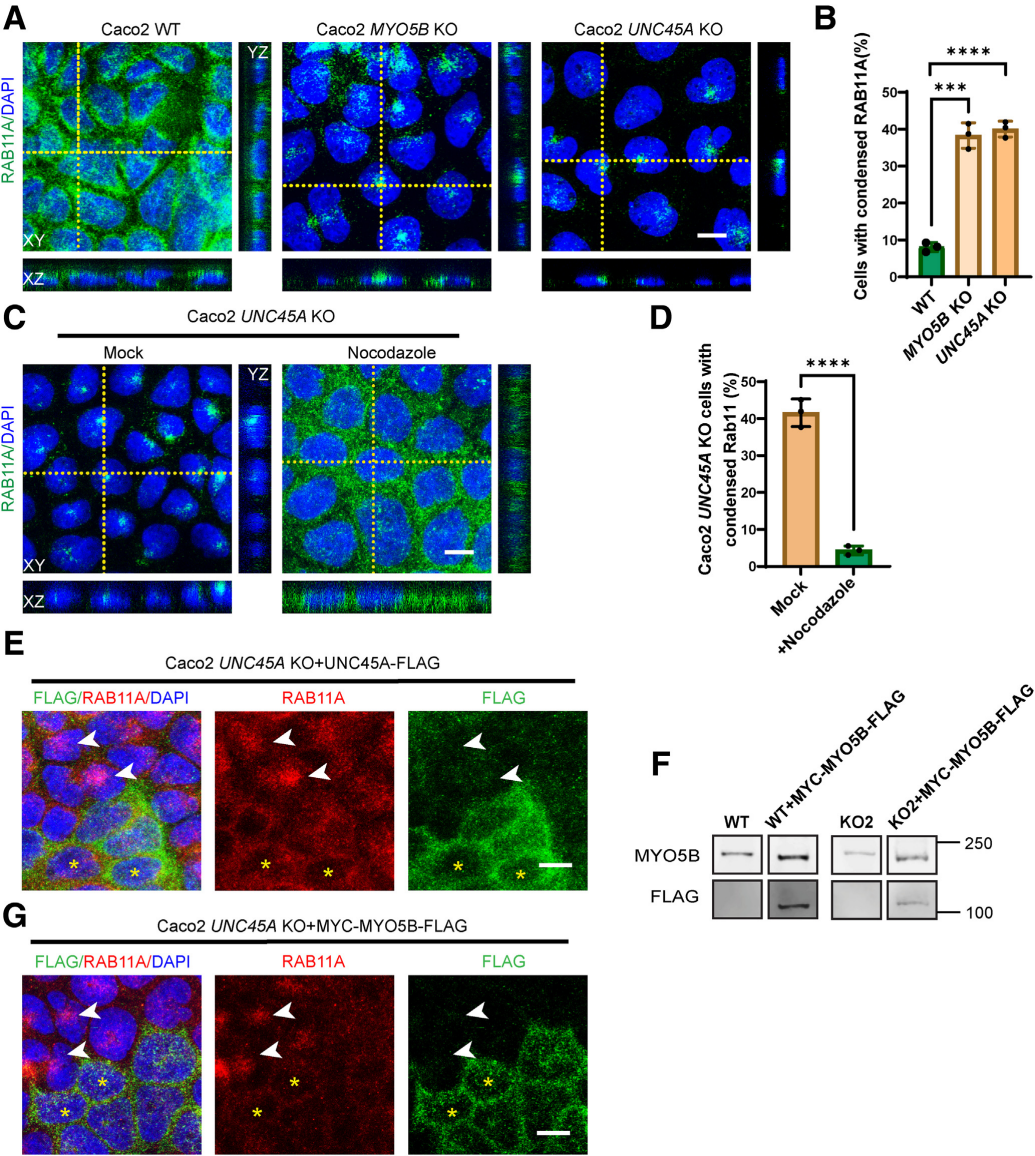
**Loss of UNC45A caused redistribution of RAB11A-positive RE in Caco-2 cells.** (*A*) Immunofluorescence microscopy image of endogenous RAB11A in WT, Caco-2 *MYO5B* KO, and *UNC45A* KO2 cells. Scale bar: 10 μm. (*B*) Quantification of percentage of condensed RAB11A in Caco-2 WT, Caco-2 *MYO5B* KO, and Caco-2 *UNC45A* KO cells. N = 3 independent experiments. Around 500 cells of 3 cell types are analyzed in each experiment. (*C*) RAB11A labeling in Caco-2 *UNC45A* KO cells and that were treated with nocodazole (33 μmol/L 1.5 hours). Scale bar: 10 μm. (*D*) Quantification of percentage of condensed RAB11A in Caco-2 *UNC45A* KO cells and treated with nocodazole. N = 3 independent experiments. More than 100 cells of 2 cell types were analyzed in each experiment. (*E–G*) RAB1A and FLAG double labelling in Caco-2 *UNC45A* KO cells re-expressing UNC45A-FLAG (*E*) and re-expressing myc-myosin Vb-FLAG (*F* and *G*). *White arrow* shows the condensed RAB11A in Caco-2 *UNC45A* KO cells, and *yellow asterisk* indicates peripheral RAB11A in Caco-2 *UNC45A* KO + UNC45A-FLAG cells. (*G*) Representative Western blot for myosin Vb and FLAG in Caco-2 WT and Caco-2 WT + myc-myosin Vb-FLAG cells, those bands come from different position of the same membrane. Representative Western blot for myosin Vb and FLAG in Caco-2 *UNC45A* KO2 and Caco-2 *UNC45A* KO2 + myc-myosin Vb-FLAG cells, those come from different positions of the same membrane. (*B*) and (*D*) Error bar indicates mean ± SD. *Black dots* indicate individual data points. *t* test, ∗∗∗∗*P* < .0001, ∗∗∗*P* < .001.

We next examined the impact of UNC45A loss on myosin Vb-dependent processes. We and others have previously shown that the loss of myosin Vb expression or function affects the spatial distribution of rat sarcoma-associated binding protein (RAB)11A-positive recycling endosomes (RE) in enterocytes in vivo^13^^,^^25^^,^^26^ and in intestinal cells in culture.^13^^,^^27^ To determine the effect of the loss of UNC45A on the spatial distribution of RAB11A-positive RE, we immunolabeled RAB11A in control and *UNC45A* KO Caco-2 cells. As shown in Figure 3*A*, RAB11A labeled vesicular structures throughout the cytoplasm and in the periphery of most control Caco-2 cells. By contrast, RAB11A showed a condensed juxta/supra-nuclear labeling pattern in *UNC45A* KO cells (Figure 3*A* and *B*). This redistribution of RAB11A-positive RE in *UNC45A* KO Caco-2 cells was similar to that observed in *MYO5B* KO Caco-2 cells (Figure 3*B*). The subcellular distribution of RAB11A-positive RE has previously been reported to depend on microtubules.^28^ Treatment of the cells with nocodazole, which is a potent microtubule cytoskeleton disrupting agent, resulted in the dispersion of RAB11-positive RE in *UNC45A* KO cells (Figure 3*C* and *D*).

To determine the causality between the loss of UNC45A and the observed redistribution of RAB11A-positive RE in *UNC45A* KO Caco-2 cells, the UNC45A-FLAG protein was reintroduced. Immunolabeling of RAB11A showed that the reintroduction of UNC45A-FLAG in *UNC45A* KO cells reduced the condensed RAB11A labeling phenotype (Figure 3*E*), which was still present in cells that did not or minimally express the UNC45A-FLAG protein (Figure 3*E*, arrows).

To determine the causative role of loss of myosin Vb in the redistribution of RAB11A-positive RE upon UNC45A depletion, a myc-FLAG–tagged human myosin Vb protein (myc-myosin Vb-FLAG) was overexpressed in *UNC45A* KO Caco-2 cells. The functionality of the encoded myosin Vb was previously demonstrated.^29^ Figure 3*F* shows the expression of myosin Vb as evidenced by Western blot analysis. In contrast to the expression of endogenous myosin Vb, the expression of myc-myosin Vb-FLAG appeared unaffected by the loss of UNC45A because expression was achieved in both control cells and *UNC45A* KO cells. Immunolabeling for RAB11A showed that introduction of myc-myosin Vb-FLAG in *UNC45A* KO cells reduced the condensed RAB11A labeling phenotype (Figure 3*G*), which was still present in cells that did not express the myc-myosin Vb-FLAG protein (Figure 3*G*, arrows).

Collectively, these results demonstrated that loss of UNC45A in Caco-2 cells reduced the expression of myosin Vb and, consequently, caused the microtubule-dependent redistribution of RAB11A-positive RE.

### Loss of UNC45A Impaired Brush Border Microvilli Organization in Caco-2 Cells

**Figure 4:**
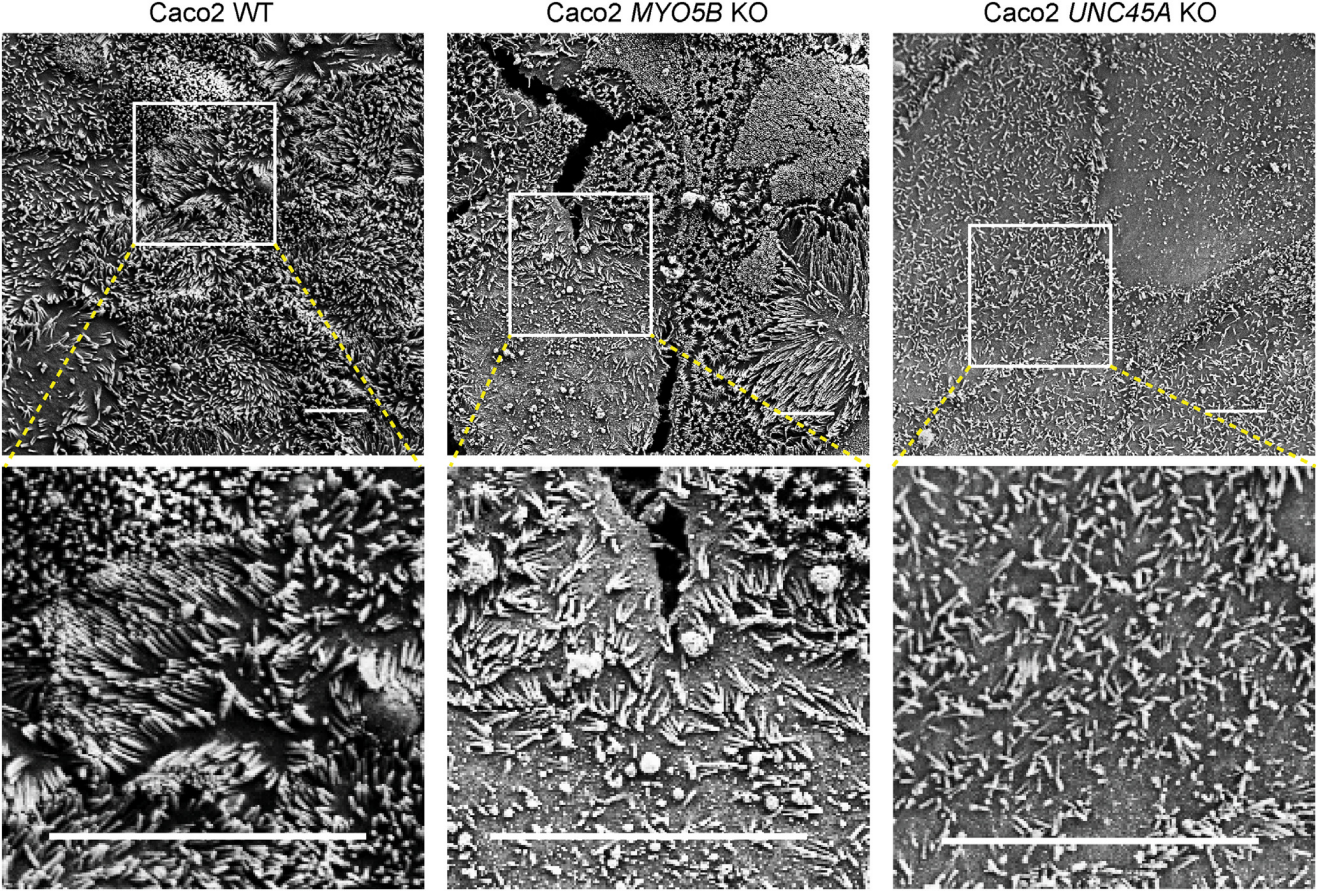
**Impaired brush border microvilli organization in Caco-2 *UNC45A* KO cells.** Scanning electron microscopy of microvilli in Caco-2 WT, *MYO5B* KO, and *UNC45A* KO2 cells. Scale bar: 5 μm.

**Figure 5:**
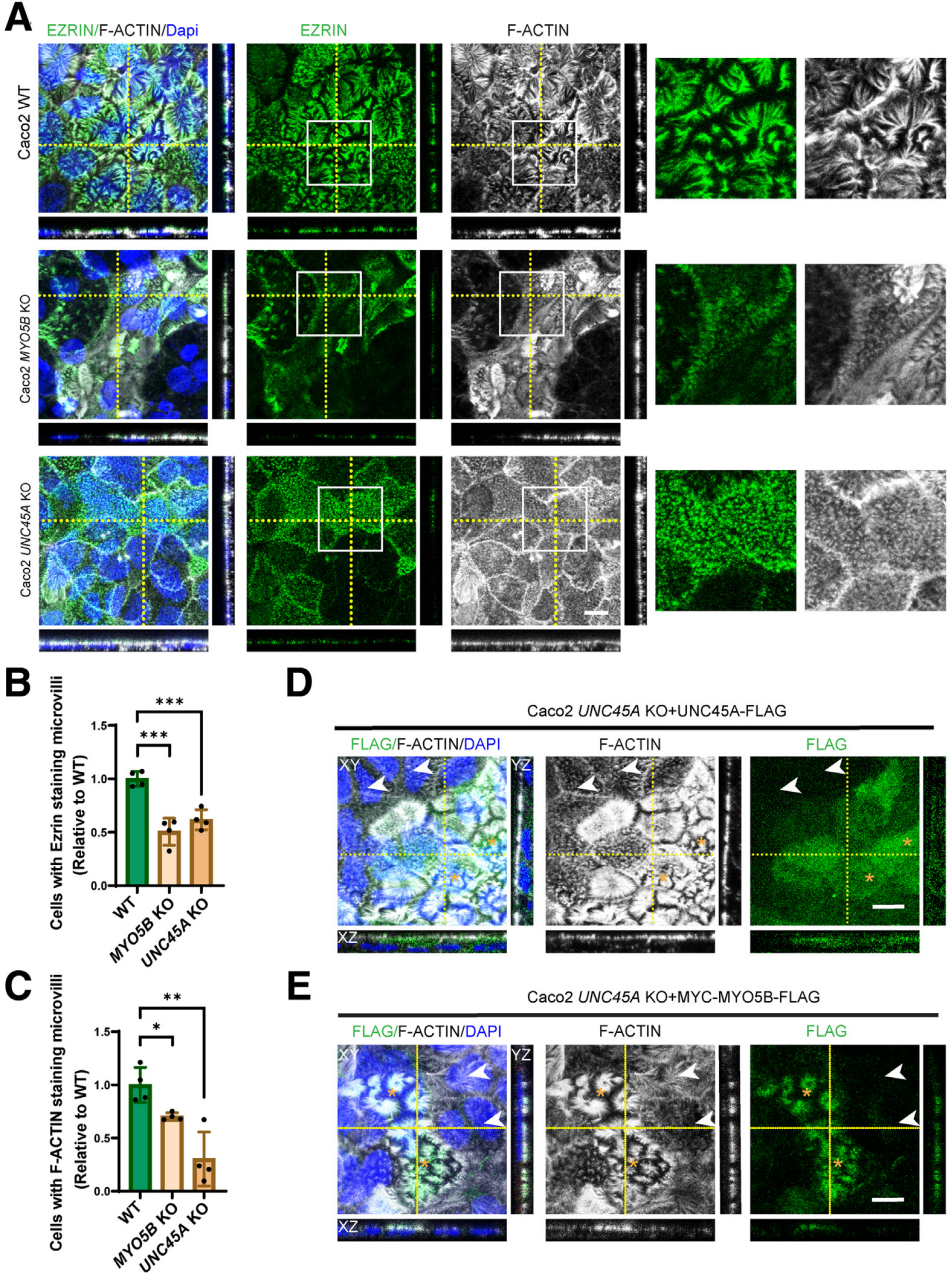
**Loss of UNC45A impaired brush border microvilli organization in Caco-2 cells.** (*A*) Immunofluorescence microscopy image of endogenous ezrin and F-actin in Caco-2 WT, *MYO5B* KO, and *UNC45A* KO2 cells. Scale bar: 10 μm. (*B* and *C*) Quantification of percentage of cells with ezrin- and F-actin–positive microvilli in Caco-2 *MYO5B* KO and Caco-2 *UNC45A* KO cells when compared with Caco-2 WT, N = 3 independent experiments. Each experiment >100 cells. Error bar indicates mean ± SD. *Black dots* indicate individual data points. *t* test, ∗∗∗*P* < .0001, ∗∗*P* < .001, ∗*P* < .01. (*D* and *E*) Ezrin, FLAG, and fluorescent phalloidin triple labelling in Caco-2 *UNC45A* KO cells re-expressing UNC45A-FLAG and re-expressing myc-myosin Vb-FLAG. *White arrow* shows reduced microvilli organization in Caco-2 *UN45A* KO cells, and yellow asterisk inidcates restored microvilli organization in Caco-2 UNC45A KO + UNC45A-FLAG cells.

The loss of myosin Vb expression and redistribution of RAB11A-positive RE have been associated with atrophic or altered organization of brush border microvilli,^8^^,^^12^^,^^13^ which is a hallmark of MVID enterocytes.^30^^,^^31^ Scanning electron microscopy, which allows for high-resolution imaging of the cells’ apical surface, revealed that microvilli in *MYO5B* KO and *UNC45A* KO Caco-2 cells were less abundant and shorter when compared with WT Caco-2 cells (Figure 4). Immunofluorescence microscopy analyses showed expression and apical localization of the brush border microvillus-associated proteins ezrin and filamentous (F-) actin in WT Caco-2 cells (Figure 5*A*). Notably, in agreement with the electron microscopy results, F-actin- and ezrin-stained microvilli at the apical surface appeared, in a patchy manner, less abundant and shorter in both *UNC45A* KO and *MYO5B* KO Caco-2 cells when compared with WT Caco-2 cells (Figure 5*A–C*). To determine the causality between the loss of UNC45A and the observed alterations in microvilli organization in *UNC45A* KO Caco-2 cells, UNC45A-FLAG was reintroduced. Immunolabeling of F-actin showed that cells that expressed UNC45A-FLAG rescued the microvilli phenotype (Figure 5*D*). The microvilli phenotype was also rescued in UNC45A KO Caco-2 cells in which myc-myosin Vb-FLAG was expressed, but not in the cells that did not express the myosin Vb protein (Figure 5*E*).

Taken together, loss of UNC45A and the subsequent reduction in myosin Vb expression resulted in less and/or less organized apical microvilli. The effect of UNC45A depletion on apical microvilli in Caco-2 cells is in agreement with the patchy brush border atrophy observed in the O2HE patient’s intestine.^5^

### Loss of UNC45A Did Not Affect the Localization of RAB11A-Positive RE and Bile Canalicular Microvilli Organization in HepG2 Cells

**Figure 6:**
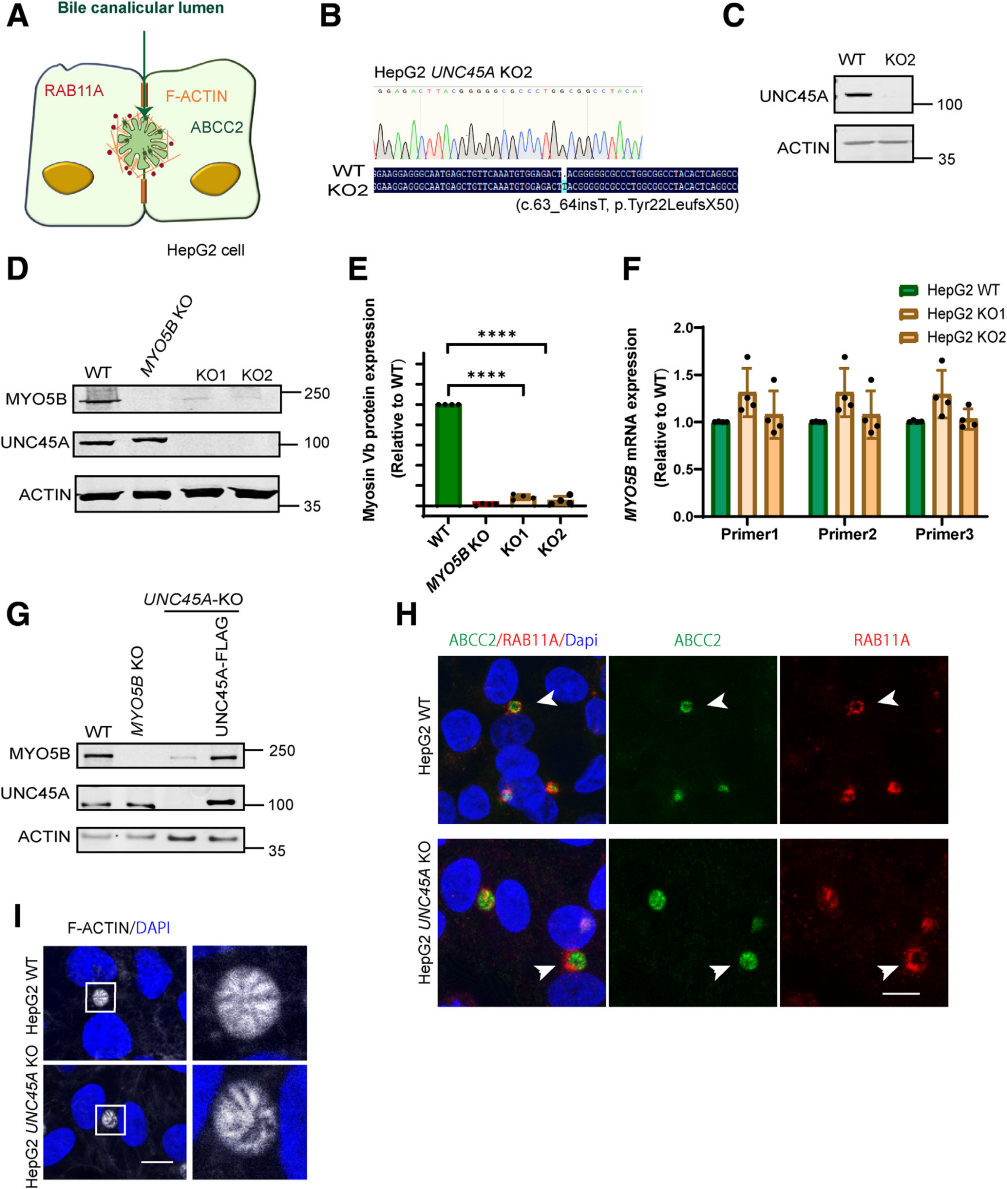
**(*A*) Cartoon illustrating the distribution of F-actin, polarized canalicular proteins ABCB11/MRP2 and RAB11A around the bile canalicular lumen in HepG2 cells.** (*B*) Sequencing chromatogram of DNA fragment targeted by CRISPR-Cas9 in HepG2 *UNC45A* KO2 cells, aligned to WT sequences. Software Chromas and DNAMAN are used to analyze sequencing results. (*C*) Representative Western blot for UNC45A in HepG2 WT and HepG2 KO2 cells. (*D*) Representative Western blot for myosin Vb and UNC45A in HepG2 WT, HepG2 *MYO5B* KO, and 2 independent HepG2 *UNC45A* KO cell lines. (*E*) Quantification of relative myosin Vb expression by Western blot in HepG2 WT and 2 independent HepG2 KO cell lines. (*F*) Quantification of the relative mRNA expression of *MYO5B* gene in 2 independent HepG2 KO cell lines. (*G*) Representative Western blot showing the expression level of MYO5B (myosin Vb) and UNC45A in HepG2 WT cell lines, MYO5B-depleted HepG2 cells lines, UNC45A-depleted cell lines and UNC45A-depleted cells lines in which the a UNC45A WT-flag gene was re-introduced. (*H*) Double labeling of ABCC2 and RAB11A in HepG2 WT and HepG2 *UNC45A* KO cells. Scale bar: 10 μm. (*I*) F-actin staining in HepG2 WT and HepG2 *UNC45A* KO cells. Error bar indicates mean ± SD. *Black dots* indicate individual data points. *t* test, ∗∗∗∗*P* < .0001.

Because *UNC45A* mutations have also been associated with intrahepatic cholestasis in some O2HE patients, we examined the effect of UNC45A depletion on myosin Vb expression in hepatocellular HepG2 cells, widely used model cell lines for the study of hepatocellular biology. HepG2 cells form bile canalicular lumens with bile canalicular surface domains that are enriched in microvilli-associated actin-filaments and bile canalicular proteins such as ABCC2 (MRP2), and which are surrounded by RAB11A-positive RE (Figure 6*A*). Gene sequencing of CRISPR-Cas9–treated HepG2 cells revealed homozygous frameshift mutations in the *UNC45A* gene in HepG2 cells (Figure 6*B*). This frameshift mutation was predicted to result in a premature termination codon and subsequent loss of expression of the UNC45A protein. Western blot analyses confirmed the absence of the UNC45A protein when compared with control cells (Figure 6*C*). *UNC45A* KO HepG2 cells (2 independent clones) showed a reduced expression of the myosin Vb protein when compared with control cells, as evidenced by Western blot (Figure 6*D*). Quantification of Western blots of 3 independent experiments revealed an 80%–100% reduction in myosin Vb protein expression in HepG2 *UNC45A* KO cells (Figure 6*E*). UNC45A mRNA expression levels were unaffected (Figure 6*F*). The reintroduction of UNC45A in UNC45A KO HepG2 cells restored myosin Vb expression levels (Figure 6*G*). Although loss of myosin Vb expression has been reported to affect the spatial distribution of RAB11A-positive RE in intestinal cells^13^ (cf, Figure 2), we recently reported that loss of myosin Vb expression in liver cells did not result in a redistribution of RAB11A-positive RE and did not affect the apical localization of the ATP Binding Cassette Subfamily C Member 2 (ABCC2), a transporter protein involved in biliary organic anion transport.^11^ In *UNC45A* KO HepG2 cells, the typical subapical localization of RAB11A, the apical localization of ABCC2, and appearance of F-actin–stained apical microvilli were likewise indistinguishable from control HepG2 cells (Figure 6*H* and *I*). Thus, although these results underscore the importance of UNC45A expression in maintaining myosin Vb expression levels, only in Caco-2 cells did the reduction in myosin Vb expression correlate with a redistribution of RAB11A-positive RE, consistent with the reported differential involvement of myosin Vb in RAB11A-positive RE positioning in these cell types.^11^^,^^13^

### The Pathogenic Mechanism of the O2HE Patient–Associated UNC45A-p.V423D Mutation

**Figure 7:**
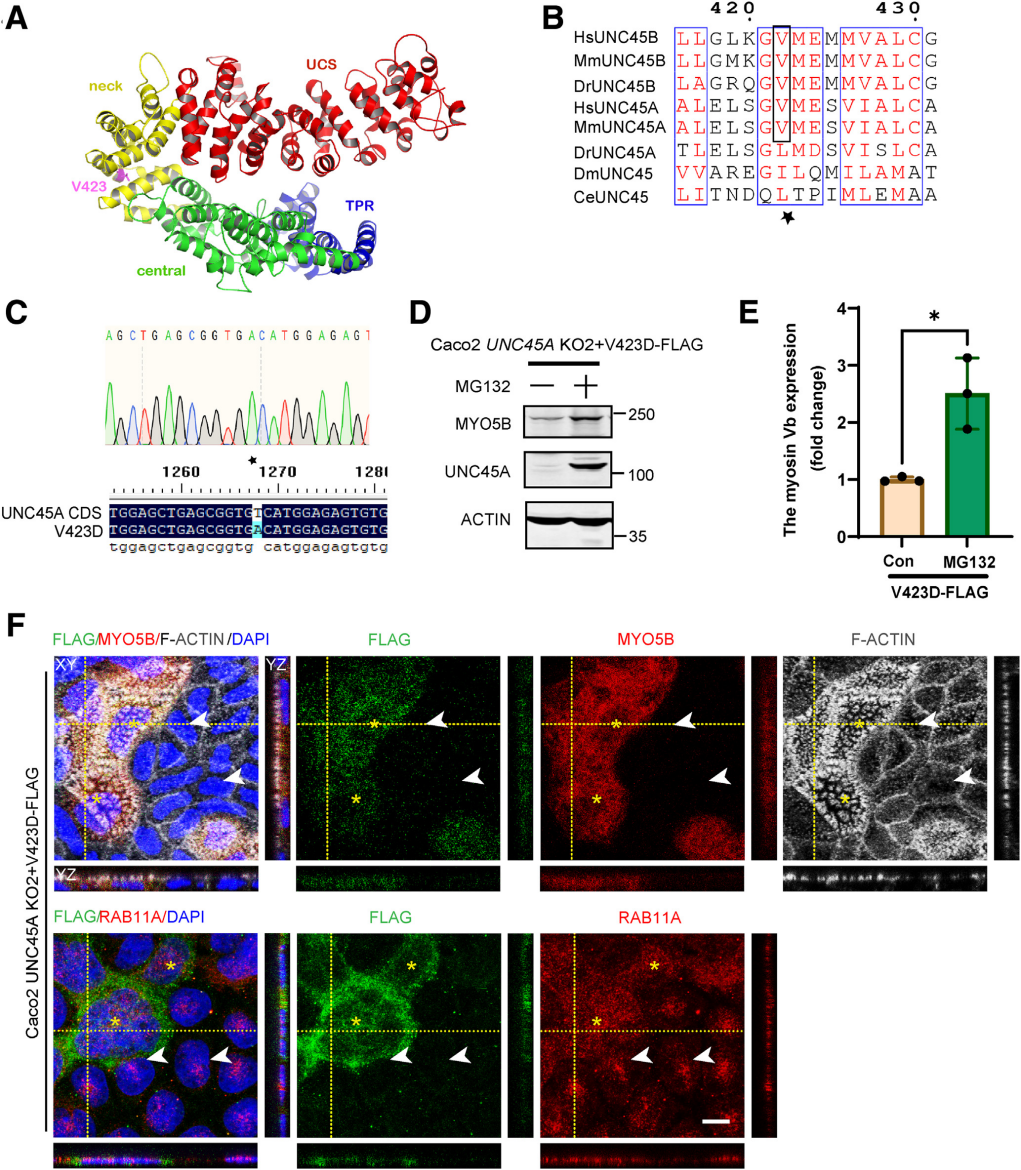
**The pathogenic mechanism of O2HE patient–associated UNC45A-p.V423D mutation.** (*A*) Sequence alignment of UNC45 proteins fragment from *Caenorhabditis elegans* (CeUNC45: G5EG62), *Drosophila melanogaster* (DmUNC45; Q960B1), *Danio rerio* (DrUNC45A: F1QU23; DrUNC45B: Q6DGE9), *Mus musculus* (MmUNC45A: Q99KD5; MmUNC45B: Q8CGY6), *Homo sapiens* (HsUNC45A: Q9H3U1-2; HsUNC45B: Q8IWX7). *Black box and star* indicate V423 mutated site. Clustal omega^27^ and ENDscript server^28^ were used to make the alignment. (*B*) Ribbon presentation of the UNC45A protein modeling based on crystal structure of UNC45 (PDB ID: c4i2wA) showing its domains and mutated site. Different colors indicate different domains explained by word with the same color. The unc45a model was built by phyre2 intensive mode.^29^ The PhyMOL software (2.5) was used to color the domains and label the mutated site. (*C*) Sequencing chromatogram of DNA fragment with mutated site in Plenti-UNC45A-p.V423D-FLAG, aligned to UNC45A cds sequence (Q9H3U1-2). Software Chromas and DNAMAN are used to analyze sequencing results. (*D*) Representative Western blot for myosin Vb and UNC45A in Caco-2 *UNC45A* KO + unc45a-p. V423D-FLAG and that treated with MG132 (5 μmol/L, 16 hours). (*E*) Quantification of relative myosin Vb expression by Western blot in Caco-2 *UNC45A* KO + unc45a-p.V423D-FLAG and that treated with MG132. N ≥ 3 independent experiments. Error bar indicates mean ± SD. *Black dots* indicate individual data points. *t* test, ∗*P* < .01. (*F*) *Upper row* shows a triple labelling of F-actin (*white*), myosin Vb (*red*), and FLAG (*green*). *Lower row* shows RAB11A (*red*) and FLAG (*green*) double labeling in Caco-2 *UNC45A* KO2 + UNC45a-p.V423D-FLAG cells. *White arrows* point to condensed RAB11A staining pattern in Caco-2 *UNC45A* KO cells, and *yellow asterisks* indicate F-actin-labeled microvilli (upper row)/peripheral RAB11A (lower row) in Caco-2 *UNC45A* KO + UNC45A-p. V423D FLAG cells. Scale bar: 10 μm.

One of the 4 O2HE patients, carrying compound heterozygous c.1268T>A and c.784C>T mutations in *UNC45A*, presented with isolated congenital intractable diarrhea with no signs of cholestasis, bone fragility, or deafness.^5^ The c.784C>T mutation is predicted to give rise to a premature termination codon at Arg262 and loss of UNC45A protein expression. The c.1268T>A mutation is predicted to cause the substitution of the hydrophobic valine (V) at amino acid position 423 in the UNC45A protein to the larger and negatively charged aspartic acid (D) (hereafter referred to as UNC45A-p.V423D). Val423 is located in the neck domain that connects the protein’s functional elements: the heat-shock protein 90-binding tetratricopeptide repeat domain and central domain at the amino-terminal side and the myosin-binding UCS domain at the carboxyl-terminal side^32^ (Figure 7*A*). Val423 is conserved in mouse UNC45A as well as in human and mouse UNC45B (Figure 7*B*). In the UNC45A orthologue of the invertebrate fruit fly *Drosophila melanogaster* and nematode *Caenorhabditis elegans,* the amino acid at this position is a leucine or isoleucine, which share the hydrophobic characteristics of valine (conservative replacement). UNC45A and UNC45B of the vertebrate zebrafish *Danio rerio* show a leucine and valine at this position, respectively, supporting that a hydrophobic branched-chain amino acid is favored at this position in the protein and that the 3 branched-chain amino acids (valine, leucine, and isoleucine) are interchangeable (Figure 7*B*).

Although predicted to be pathogenic, the mechanism that underlies the pathogenicity of the p.V423D mutation is not known. Here we used site-directed mutagenesis to introduce the c.1268T>A variant in human *UNC45A* (Figure 7*C*) and show by means of Western blot analyses that the reintroduction of the *UNC45A-*c.1268T>A variant in *UNC45A* KO Caco-2 cells (Figure 7*D*) resulted in very little if any rescue of UNC45A expression and, accordingly, did not rescue full-length myosin Vb protein expression (Figure 7*D*). MG132 treatment of *UNC45A* KO Caco-2 cells in which the *UNC45A*-c.1268T>A variant was reintroduced significantly increased the expression of a full-length (∼103 kDa) UNC45A-p.V423D (Figure 7*D* and *E*). This indicated that this mutant protein was fully formed but was unstable and degraded by the proteasome. Moreover, MG132 treatment of *UNC45A* KO Caco-2 cells in which FLAG-tagged UNC45A-p.V423D was re-expressed (Figure 7*D* and *E*) but not of *UNC45A* KO as such (cf, Figure 2*B*) and increased the expression level of myosin Vb. Interestingly, when examining UNC45A-p.V423D expression by immunofluorescence microscopy, we noted that although UNC45A-p.V423D was not visibly expressed in most cells that were not treated with MG132, a small number of cells did express the FLAG-tagged UNC45A-p.V423D (Figure 7*F*, green). Notably, these FLAG-positive cells also showed increased expression of myosin Vb (Figure 7*F*, red) and a rescue of the microvilli phenotype as evidenced by F-actin staining (Figure 7*F*, white). FLAG-positive cells (Figure 7*F*, lower panel, green) also showed a rescue of RAB11A-positive RE distribution (Figure 7*F*, lower panel, red). We conclude that (1) the c.1268A>T-encoded p.V423D mutation in the UNC45A protein caused its instability and subsequent proteasomal degradation, and (2) the p.V423D mutation, as such, did not impair the function of the UNC45A protein with regard to its ability to promote myosin Vb expression, RAB11A-positive RE positioning, and microvilli development.

## Discussion

In this study we tested the hypothesis that UNC45A and myosin Vb, the losses of which are associated with 2 rare diseases with shared enteropathy,^5^^,^^8^ are functionally linked. We demonstrated that loss of expression of O2HE syndrome–associated UNC45A or replacement of the endogenous *UNC45A* gene with an *UNC45A* gene carrying the c.1268T>A mutation in intestinal and liver epithelial cell lines resulted in a significant reduction in the expression of MVID-associated myosin Vb. We demonstrated that the patient’s p.V423D substitution in UNC45A^5^ resulted in an unstable and rapidly degraded mutant UNC45A-p.V423D protein resulting in loss of its expression. Causality was confirmed by the observed restoration of myosin Vb expression in UNC45A-depleted cells after the re-expression of UNC45A.

In vitro transcription/translation-coupled assays suggested that UNC45A regulated the folding of the smooth muscle myosin motor domain post-translationally.^1^ In our study, the reduction in myosin Vb protein expression upon UNC45A depletion was not accompanied by a reduction of myosin Vb mRNA, indicating that *MYO5B* gene transcription was not affected. Inhibition of proteasome activity did not restore full-length myosin Vb expression in UNC45A-depleted cells, as evidenced by Western blot analysis. Because the anti-myosin Vb antibody recognizes an epitope that is located in the coiled-coil domain (N1054-K1148; UniProt Identifier# Q9ULVO-1) that is distal to the motor domain, we could not determine whether an incompletely synthesized myosin Vb protein was formed. The expression of an exogenous, amino-terminal myc-tagged myosin Vb could not be used to detect myc-positive myosin Vb (motor domain) fragments in UNC45A–depleted cells because its expression appeared insensitive to UNC45A depletion. Thus, our results support a role for UNC45A in the regulation of myosin Vb at the protein level. Additional studies, which are beyond the scope of this study, are needed to unravel the precise mechanism via that UNC45A controls endogenous myosin Vb expression.

We demonstrated that prohibiting endogenous *UNC45A* gene expression in intestinal cells not only reduced myosin Vb protein expression but also affected 2 myosin Vb-dependent processes, microvilli development and RAB11A-positive RE positioning. Both processes have been implicated in the pathogenesis of MVID.^12^^,^^13^^,^^19^ Previous studies demonstrated that the spatial positioning of RAB11A-positive RE is controlled by cytoskeletal factors. For example, myosin Vb mutants that lacked the actin-binding domain caused the accumulation of RAB11A-positive RE in the juxta-nuclear region near the microtubule-organizing center,^28^ similar as seen in MVID enterocytes in vivo.^13^ Disruption of the microtubule network with nocodazole, by contrast, caused the dispersion of RAB11A-positive RE toward the cell periphery.^28^ A model has been proposed where myosin V motors participate in a “tug-of-war” with microtubule minus end- (ie, microtubule organizing center-directed) dynein motors.^33^ The nocodazole-sensitive juxta-nuclear accumulation of RAB11A-positive RE in *UNC45A*-depleted Caco-2 cells is in line with such a model.

Notably, UNC45A has been identified as a microtubule-destabilizing protein,^34^^,^^35^ and depletion of UNC45A may have elicited a microtubule-stabilizing effect. Relevant in this context is that treatment of a kidney epithelial cell line with the microtubule-stabilizing drug taxol also caused a redistribution of RE, albeit different from the effects of myosin Vb mutants.^28^ Whether potential changes in microtubule dynamics may have contributed to the redistribution of RAB11A-positive RE in UNC45A-depleted Caco-2 cells, as shown in this study, remains to be investigated. Regardless, our observation that reintroduction of WT myosin Vb in UNC45A-depleted Caco-2 cells restored the spatial distribution of RAB11A-positive RE indicated that the effect of UNC45A depletion on RE distribution was predominantly via myosin Vb.

The observed reduced myosin Vb expression and RE positioning could not yet be validated in O2HE patient intestinal tissue because of lack of access to patient material. Nonetheless, Caco-2 and HepG2 cells have been proven to be highly predictive models for the cellular phenotypes in MVID(-like) disease. Indeed, the depletion of myosin Vb, syntaxin-3, or syntaxin-binding protein-2 in Caco-2 cells faithfully reproduced the key in vivo phenotypes of intestinal epithelial cell in MVID patients resulting from mutations in *MYO5B,*^12^^,^^13^^,^^19^^,^^27^^,^^36^ *STX3,*^20^^,^^37^ and *STXBP2,*^18^^,^^37^^,^^38^ with equal performance as human and mouse 3D enteroids.^20^^,^^24^^,^^38^^,^^39^ The functional relationship between these 3 genes in MVID was also reproduced in Caco-2 cells.^18^^,^^22^ In addition, myosin Vb-depleted Caco-2 and HepG2 cells faithfully reproduced the in vivo phenotypic differences between intestinal and liver cells, respectively, seen in *myo5b* KO mice.^11^ Moreover, both *UNC45A*-depleted Caco-2 and HepG2 cells showed reduced myosin Vb expression while preserving these downstream in vivo phenotypic differences between intestinal and liver cells (this study), which underscores that these phenotypes are not cell line anomalies and predictive of the in vivo situation. Finally, the microvillus atrophy as observed in UNC45A-depleted Caco-2 cells is in agreement with the reported microvillus abnormalities in the enterocytes of the O2HE patient intestine.^5^ Therefore, the cell lines used in this study can be considered telling models and reliable alternatives when patient material is not (yet) available.

The unidirectional link between UNC45A and myosin Vb expression as demonstrated in this study suggests that reduced expression of myosin Vb and perturbation of myosin Vb-dependent processes may underlie (part of) the intestinal symptoms in O2HE patients. Furthermore, as a regulator of myosin Vb expression, changes in the expression or functionality of UNC45A may contribute to the heterogeneity and complexity of clinical presentations associated with microvillus inclusion disease.

O2HE patients also display heterogeneity in the clinical presentation. Three of the 4 O2HE patients described by Esteve et al^5^ displayed intractable diarrhea, all requiring parenteral nutrition. In one of these patients (patient B.II.4^5^) the diarrhea resolved at unspecified later age, and her sister (patient B.II.3^5^) reportedly did not present with diarrhea. The reason for this heterogeneity in diarrhea presentation is not clear but is also observed in *MYO5B*-associated MVID.^40^ Possibly, patient-specific *UNC45A* mutations determine the diarrheal phenotype in O2HE. For example, the siblings B.II.4 and B.II.3^5^ carried mutations located in the very C-terminal end of the armadillo-like domain of UNC45A, which has different functions when compared with the N-terminal tetratricopeptide repeat domain and/or central domain of UNC45A in which the mutations of the other 2 patients were identified. The reported difference in diarrhea presentation between siblings B.II.4 and B.II.3^5^ further suggests that also non-genetic factors may play a role.

In contrast to intestinal Caco-2 cells, little if any spatial redistribution of RAB11A-positive RE or altered appearance of bile canalicular membrane microvilli was observed after depletion of UNC45A in hepatic HepG2 cells. This is in agreement with our previous studies in which we demonstrated that loss of myosin Vb expression in HepG2 cells, human pluripotent stem cell-derived hepatocyte-like cells, and mouse hepatocytes in vivo did not result in the mislocalization of RAB11A-positive RE.^11^ This is also supported by patient data that show that in contrast to missense *MYO5B* mutations, bi-allelic mutations in the *MYO5B* gene that are predicted to result in the loss of myosin Vb expression have not been identified in patients with isolated intrahepatic cholestasis.^9^^,^^10^^,^^41^ Notably, the O2HE patient with the UNC45A-p.V423D mutation did not display intrahepatic cholestasis.^5^ We do not know whether the *UNC45A* mutations reported in O2HE patients who did present with cholestasis will lead to a similar loss of myosin Vb expression and whether any myosin Vb remaining will function properly in the absence of UNC45A or may behave as a dysfunctional/mutant-like myosin Vb. It is also possible that *UNC45A* mutations affect other myosin proteins that have been proposed to play a role in bile acid secretion (eg, myosin II^42^). Future studies that address the effects of *UNC45A* mutations from cholestatic O2HE patients and the UNC45A clientele are expected to shed further light on geno-/phenotype correlations.

We demonstrated that the *UNC45A*-c.1268T>A variant caused instability and proteasomal degradation of the mutant UNC45A-p.V423D protein. In agreement, Esteve et al^5^ did not observe a rescue of the intestinal phenotype upon introduction of the p.V423D mutant in *unc45a*-depleted zebrafish. When proteasomal degradation was prevented in Caco-2 cells, the resultant expression of UNC45A-P.V423D (replacing the endogenous UNC45A) did not ablate the function of the mutant UNC45A protein with regard to its ability to promote myosin Vb expression and microvilli development. In support of this, V423 is not located in the functional tetratricopeptide repeat or UCS domains of the UNC45A protein, and valine (similar to leucine and isoleucine) is assumed to be non-reactive and as a hydrophobic branched-chain amino acid more likely to be involved in protein folding and structure.^43^ These results explain the mechanism underlying the pathogenicity of the UNC45A-p.V423D mutation and suggest that a pharmacologic intervention aimed at stabilizing UNC45A-p.V423D protein expression in the intestine may restore intestinal function.

Taken together, this study revealed a functional relationship between UNC45A and myosin Vb protein expression, thereby connecting 2 rare congenital diseases with overlapping intestinal symptoms at the molecular level. Protein instability rather than functional impairment underlies the pathogenicity of the O2HE syndrome–associated UNC45A-p.V423D mutation.

## Methods

### Cell Culture

Human Caco-2 cells (American Type Culture Collection HTB-37), HepG2 cells (American Type Culture Collection HB8065), and human embryonic kidney (HEK)293 cells (American Type Culture Collection CRL-1573) (American Type Culture Collection, Gaithersburg, MD) were cultured in high-glucose Dulbecco modified Eagle medium (catalogue number 11965084; Thermo Scientific Fisher, Waltham, MA) supplemented with 10% heat-inactivated fetal calf serum (Invitrogen, Waltham, MA), 2 mmol/L L-glutamine and antibiotics (penicillin 100 IU/mL, streptomycin 100 μg/mL) (Thermo Scientific Fisher), and incubated at 37°C in a humidified atmosphere with 5% CO_2_ for Caco-2 and Hek293 and 7.5% CO_2_ for HepG2. For experiments, cells were grown on poly-L-lysine–coated coverslips and fixed after 3 days or 14 days in culture.

### Antibodies and Dyes

Antibodies used in this study were ABCC2/ MRP2 (catalogue number MAB4150; Millipore, Burlington, MA), RAB11A (mouse) (catalogue number 610656; BioScience, Franklin Lakes, NJ), RAB11A (rabbit) (catalogue number ab128913; Abcam, Cambridge, MA), myosin Vb (catalogue number NBP1-87746; Novus), FLAG (catalogue number F3165; Sigma-Aldrich, St Louis, MO), UNC45A (rabbit) (catalogue number HPA039228; Merck, Kenilworth, NJ), UNC45A (mouse) (catalogue number ADI-SRA-1800-F; Enzo Life Sciences, Farmington, NY), beta-tubulin (catalogue number T4026; Merck), c-myc (catalogue number 631206; Takara, Kusatsu, Japan), ezrin (catalogue number SC58758; Santa Cruz Biotechnology, Dallas, TX), beta-actin (catalogue number A5441; Sigma-Aldrich), syntaxin-3 (catalogue number ab133750; Abcam), and munc18-2 (catalogue number ab103976; Abcam). Fluorescently labeled phalloidin (catalogue number A22284) was from Invitrogen.

### Oligonucleotides for Plasmid Construction

MYC deletion forward 1: GCAGCAAATGATATCCTG, MYC deletion reverse 1: CTCTCCATCTTGGTTGGG, deletion forward 2: GATTACAAGGATGACGACGATAAGGTTTAA, MYC deletion reverse 2: GAGCGGCCGCGTACGCGT, UNC45A p. Val423Asp forward: CTGAGCGGTGaCATGGAGAGTGTG, UNC45A p.Val423Asp reverse: CTCCAAGGCCCGGTTGCC, UNC45A CRISPR 1 forward: CACCGAGACTACGGGGGCGCCCTGG, UNC45A CRISPR 1 reverse: AAACCCAGGGCGCCCCCGTAGTCTC, UNC45A CRISPR 2 forward: CACCGGTTCAAATGTGGAGACTACG, UNC45A CRISPR 2 reverse: AAACCGTAGTCTCCACATTTGAACC. Caco-2 UNC45A KO2 and HepG2 UNC45A KO2 were both generated with oligos UNC45A CRISPR 2 forward and UNC45A CRISPR 2 reverse.

### Plasmids

Full-length tagged human *UNC45A* sequence was amplified from plasmid pCMV6-Entry-UNC45A (Origene RC206953) through polymerase chain reaction. Amplified UNC45A-myc-FLAG was inserted into pENTR1a vectors, and then the myc tag was deleted. Patient-derived point mutations were produced on the myc tag deleted pENTR1a plasmid. The Q5 Site-Directed Mutagenesis Kit (catalogue number E0554S; New England Biolabs, Ipswich, MA) with primers designed in the NEBaseChanger tool was used for myc tag deletion and point mutation creation. UNC45A-FLAG and mutant UNC45A-FLAG were cloned into lentiviral vectors.^11^ Plenti-myc-Myosin Vb has been described.^29^ According to protocols attached with plentiCRISPR-V2 vector (Addgene #52961, Watertown, MA), 2 CRISPR plasmids were constructed against the second exon of *UNC45A*.

### Virus Production and Transduction

With a second-generation system based on pCMVdR8.1 and pVSV-G, lentiviral particles were generated to transduce cells. For virus production, 1 × 10^6^ HEK293T were plated in a well of a 6-well plate. Vectors of 1200 ng pLenti-plasmid, 1000 ng pCMVdR8.1, and 400 ng pVSV-G were mixed into 100 μL OptiMEM with 7.8 μL Fugene (catalogue number E3211; Promega, Madison, WI). After 5 minutes of incubation, the mixture was added into the medium of HEK293T. The next morning the medium was removed, and virus production medium: DMEM with 10% fetal calf serum and Dulbecco modified Eagle medium: F12 (no serum) in the ratio of 1:3 was added. After a 2-day incubation the virus-containing supernatant was filtered through a polyvinylidene difluoride membrane–based 0.45 μm filter (GE Healthcare, Hatfield, UK) and harvested as viral particles. After seeding in 6-well plate at least for 4 hours, the target cells were cultured with 500 μL viral particles and 1 mL medium supplemented with 8 μg/mL polybrene. After overnight incubation, the virus-containing medium was replaced with antibiotics-containing medium (2.5 μg/mL puromycin, 4 μg/mL blasticidin).

### Western Blot

Cell pellets were lysed with lysis buffer (150 mmol/L NaCl, 0.5% non-iodet P40, 0.5 Mm ethylenediamine tetraacetic acid, 10 mmol/L trisaminomethane (Tris; pH 7.5) supplemented with protein inhibitors (Roche, Almere, the Netherlands). Bicinchoninic acid (Sigma B-9643) and 4% (w/v) CuSO4.5H2O were used to determine protein concentration. Equal amounts of protein lysates were mixed with sample buffer 2% sodium dodecyl sulfate, 5% β-mercaptoethanol, 0.125 mol/L Tris–HCl, pH 6.8, 40% glycerol, 0.01% bromophenol blue) and heated at 95°C for 5 minutes and resolved by sodium dodecyl sulfate-polyacrylamide gel electrophoresis and then transferred onto polyvinylidene difluoride membranes. The membranes were blocked with Odyssey-blocking buffer (LI-COR, Lincoln, NE) and incubated with primary antibody for 2 hours at room temperature or overnight at 4°C and secondary antibody for 2 hours at room temperature. Blots were then detected by Odyssey Imaging System (LI-COR) and quantified with Odyssey software.

### Immunofluorescence Microscopy

Cells were fixed with 4% paraformaldehyde for 20 minutes and permeabilized in 0.2% (v/v) Triton X-100 for 10 minutes at room temperature and then blocked in phosphate-buffered saline solution containing 1% (w/v) fetal calf albumin in 37°C incubator for more than 1 hour. Cells were incubated with primary antibodies overnight at 4°C, and secondary antibodies, 4′,6-diamidino-2-phenylindole (DAPI), Alexa-633–labeled phalloidin for 2 hours at room temperature. Coverslip were mounted in Dako mounting medium and dried overnight. All images shown in figures were taken by a SP8X DLS confocal (Leica, Wetzlar, Germany), 63×, oil-immersion objective and analyzed using a combination of ImageJ and Adobe Photoshop. x-y mages are shown as maximum intensity projections. Yellow lines indicate position of x-z, y-z sections.

### Scanning Electron Microscopy

Cells grown on glass coverslips were fixed by adding 1 volume 2% glutaraldehyde in 0.1 mol/L sodium cacodylate to the cells still in culture medium. After 10 minutes this mixture was replaced by pure fixative for another 20 minutes. Cells were washed with 0.1 mol/L sodium cacodylate and post-fixed in 1% osmium tetroxide in 0.1 mol/L sodium cacodylate for 1 hour, followed by 3 times washing with water. Cells were dehydrated with ethanol (10 minutes 70%, 10 minutes 100%, 20 minutes 100%, 30 minutes 100%) and dried using critical point drying from carbon dioxide. Samples were glued on stubs using double-sided carbon adhesives and sputter coated with 10 nm chrome before imaging in a Zeiss Supra55 SEM (Oberkochen, Germany) at 3 KV.

### Real-Time Quantitative Polymerase Chain Reaction

As described,^11^ RNA was extracted by trizol reagent (Sigma-Aldrich) at room temperature. Complementary DNA was synthesized from RNA in the presence of oligo (dT) 12-18 (Invitrogen) and deoxyribonucleotide triphosphate (Invitrogen) in a reaction catalyzed by Moloney murine leukemia virus reverse transcriptase (Invitrogen). Relative *MYO5B* expression level was determined by real-time quantitative polymerase chain reaction using ABsolute aPCR SYBR Green Master Mix (Westburg, Utrecht, Netherlands) in a Step-One Plus Real-Time PCR machine (Applied Biosystems, Waltham, MA). Primers to target *MYO5B* sequences were TCGGGGTCCTGGACATCTAT (qRT1 forward), CCAGTTTGAAAACATGCGAGTTG (qRT1 reverse), TTGGAAGTGTGGCGATTCAG (qRT2 forward), GCAGTCGGCAGAAGTTGCTT (qRT2 reverse), TCCCCTGAATGAATTTGAAG (qRT3 forward), GGTCATTCCGCTCTTGTAGT (qRT3 reverse).

### Statistics

All experiments were performed at least 3 times independently. Two-tailed unpaired Student *t* tests were performed to determine statistical significance with Graph Pad Prism software (San Diego, CA). *P* values were ∗*P* < .05, ∗∗*P* < .01, and ∗∗∗*P* < .001.

## CRediT Authorship Contributions

Qinghong Li (Conceptualization: Lead; Data curation: Lead; Formal analysis: Equal; Funding acquisition: Lead; Investigation: Equal; Methodology: Lead; Project administration: Equal; Writing – original draft: Equal; Writing – review & editing: Equal)

Zhe Zhou (Formal analysis: Equal; Investigation: Equal; Writing – original draft: Supporting; Writing – review & editing: Equal)

Yue Sun (Formal analysis: Equal; Investigation: Equal; Methodology: Supporting; Writing – original draft: Supporting; Writing – review & editing: Equal)

Chang Sun (Formal analysis: Equal; Investigation: Equal; Writing – original draft: Supporting; Writing – review & editing: Equal)

Karin Klappe (Formal analysis: Equal; Investigation: Equal; Methodology: Equal; Project administration: Equal; Writing – original draft: Supporting; Writing – review & editing: Equal)

Sven van Ijzendoorn (Conceptualization: Lead; Formal analysis: Equal; Investigation: Equal; Methodology: Equal; Project administration: Equal; Supervision: Lead; Writing – original draft: Equal; Writing – review & editing: Equal)
